# H7N9 bearing a mutation in the nucleoprotein leads to increased pathology in chickens

**DOI:** 10.3389/fimmu.2022.974210

**Published:** 2022-10-06

**Authors:** Daniel S. Layton, Jeffrey Butler, Cameron Stewart, Vicky Stevens, Jean Payne, Christina Rootes, Celine Deffrasnes, Som Walker, Songhua Shan, Tamara J. Gough, Christopher Cowled, Kerri Bruce, Jianning Wang, Katherine Kedzierska, Frank Y. K. Wong, Andrew G. D. Bean, John Bingham, David T. Williams

**Affiliations:** ^1^ Commonwealth Scientific and Industrial Research Organisation (CSIRO) Health and Biosecurity, Australian Centre for Disease Preparedness, Geelong, VIC, Australia; ^2^ Commonwealth Scientific and Industrial Research Organisation (CSIRO) Australian Animal Health Laboratory, Australian Centre for Disease Preparedness, Geelong, VIC, Australia; ^3^ Department of Microbiology & Immunology, University of Melbourne, at the Peter Doherty Institute for Infection & Immunity, Parkville, VIC, Australia

**Keywords:** interferon, influenza, Mutation, T cell, H7N9

## Abstract

The zoonotic H7N9 avian influenza (AI) virus first emerged in 2013 as a low pathogenic (LPAI) strain, and has repeatedly caused human infection resulting in severe respiratory illness and a mortality of ~39% (>600 deaths) across five epidemic waves. This virus has circulated in poultry with little to no discernible clinical signs, making detection and control difficult. Contrary to published data, our group has observed a subset of specific pathogen free chickens infected with the H7N9 virus succumb to disease, showing clinical signs consistent with highly pathogenic AI (HPAI). Viral genome sequencing revealed two key mutations had occurred following infection in the haemagglutinin (HA 226 L>Q) and nucleoprotein (NP 373 A>T) proteins. We further investigated the impact of the NP mutation and demonstrated that only chickens bearing a single nucleotide polymorphism (SNP) in their IFITM1 gene were susceptible to the H7N9 virus. Susceptible chickens demonstrated a distinct loss of CD8^+^ T cells from the periphery as well as a dysregulation of IFNγ that was not observed for resistant chickens, suggesting a role for the NP mutation in altered T cell activation. Alternatively, it is possible that this mutation led to altered polymerase activity, as the mutation occurs in the NP 360-373 loop which has been previously show to be important in RNA binding. These data have broad ramifications for our understanding of the pathobiology of AI in chickens and humans and provide an excellent model for investigating the role of antiviral genes in a natural host species.

## Introduction

In March 2013 in China, a low pathogenic novel reassortant subtype H7N9 avian influenza (AI) virus emerged in human patients presenting with fever and lower respiratory tract symptoms. During subsequent annual epidemics there have been >1500 human infections and >600 deaths (1–3). Unlike other zoonotic AI viruses that are capable of causing high mortality in humans, low pathogenic AI (LPAI) H7N9 causes no discernible disease in chickens and other poultry, despite being able to efficiently replicate in these species, making detection and eradication difficult (4–6), To date, H7N9 human infections have predominantly been associated with close contact with live poultry and/or contaminated environments, suggesting limited human-to-human transmission (7). Transmission has been demonstrated in ferrets; however, a high degree of variability exists in transmission, which appears strain dependant (8, 9). This variation implies that host factors and/or host-pathogen interactions play a key role in disease spread and outcome. Mutations leading to higher transmissibility and/or pathology may present serious risk of pandemic spread. Therefore, investigating H7N9 host-pathogen interactions are vital to our ability to prevent and control further outbreaks.

As with other AI, disease severity as well as transmission potential is likely to result from both viral and host factors (10). Human H7N9 infections present with hallmark characteristics of severe AI virus infection, including lymphopenia and hypercytokinemia (4). It has been demonstrated that early and persistent hypercytokinemia, in particular when it involves IL-6, IL-8, and MIP-1β, is predictive of poor patient outcomes (11). Furthermore, hypercytokinemia has been linked to a well-known host influenza susceptibility factor, IFITM3 dysregulation, resulting from the rs12252-C genotype (11, 12). Recovery from acute H7N9 infections have been attributed to early recruitment of host lymphocytes, in particular T cells, whereas patients who succumb to the disease often had little or no lymphocyte activity (13). In addition to host factors, viral attributes also play a key role in susceptibility and pathology of different species. For H7N9 it has been demonstrated that the HA protein is predominantly adapted for binding the human sialic acid as they contain V186 and L226, which are crucial for viral attachment and entry (14). Additionally, the virus has been shown to obtain further fitness through well characterised E627K and D701N mutations of the PB2 gene (15, 16). In 2017, a strain of H7N9 that incorporated a multiple basic cleavage site (MBCS) and converted to HPAI was identified circulating in China and demonstrated high pathogenicity in chickens (17, 18). These differences in host susceptibility and viral fitness give great insight into the mechanisms of differential pathogenesis and offer the potential to identify mechanisms for targeted therapy and vaccination.

We report here the identification and characterisation of a novel mutation in the H7N9 NP that leads to lethal infection in chickens in the absence of a MBCS. This NP mutation occurs in a well described immunodominant antigenic epitope, suggesting antigen presentation may play a role in increased pathogenesis. Furthermore, we have identified a SNP in chicken IFITM1 that strongly correlates with disease outcome. This work adds vital information to host-pathogen interactions required for increased susceptibility and severe disease.

## Results

### H7N9 LPAI caused severe disease in SPF chickens

**Figure 1:**
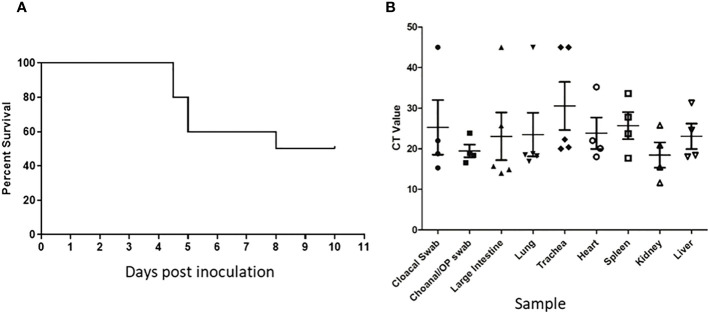
Unexpected deaths following H7N9 infection. Survival curve **(A)** and RT-qPCR detection of H7N9 systemic infection **(B)** following ONO inoculation of SPF chickens with A/Anhui/1/13 (H7N9). **(A)** Following inoculation of ten 16-week old SPF chickens with 10^7.7^ EID_50_ of A/Anhui/1/13 (H7N9), five chickens either died or were humanely killed between day 5 and 8 pi. **(B)** Swab and tissue samples taken upon necropsy from the five chickens that died/were humanely killed, were subjected to RT-qPCR analysis of the influenza M gene to assess the degree of systemic infection in the affected chickens.

**Figure 2:**
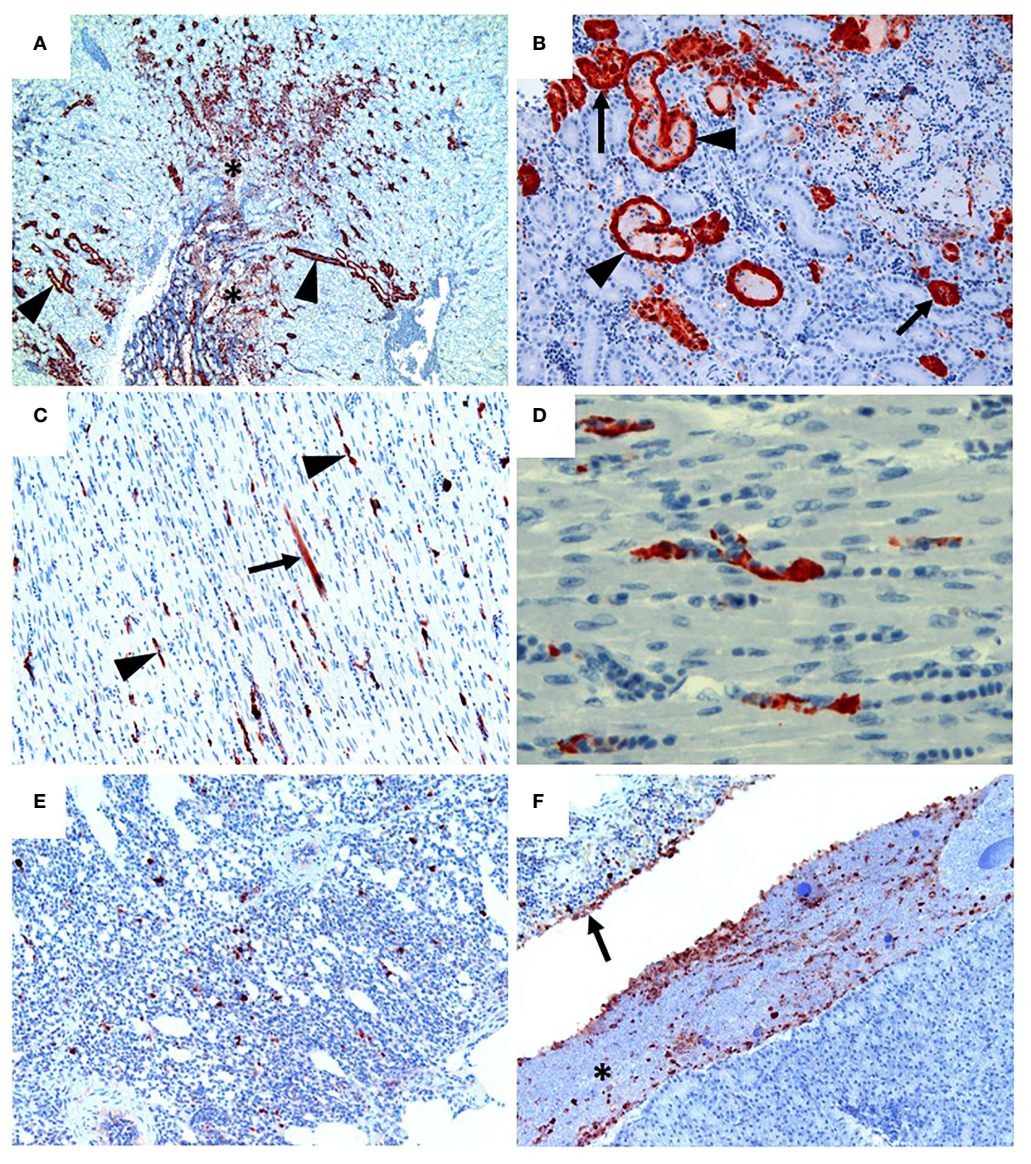
H7N9 avian influenza viral localisation in tissues of 16 week-old chickens inoculated *via* the ONO route. **(A)** Kidney (Chicken 11), showing viral antigen in renal tubules (arrowheads). Viral infection is also associated in some regions with localised necrosis of renal interstitium and tubules (*). **(B)** Kidney (Chicken 11), higher magnification of the same tissue, showing viral antigen in the epithelium of the tubules. While some tubules appear healthy (arrows), others are distended with necrotic material (arrowheads). **(C)** Heart (Chicken 11), showing that viral antigen is mainly within capillary endothelium (arrowheads) and in one cardiomyocyte (arrow). **(D)** Heart (Chicken 11), higher magnification of the same tissue, showing detail of viral antigen in capillaries. Enucleated erythrocytes can be seen lined up within some of the capillaries. **(E)** Lung (Chicken 5), showing viral antigen within single cells, presumed to be macrophages, within the lung parenchyma. **(F)** Viral antigen is present in the yolk material (*) and inflammatory exudate within the airsac overlying the pancreas (bottom right of the image) and in the airsac membrane overlying the inflamed serosal surface of the duodenum (arrow) (Chicken 11). Immunohistochemistry for influenza nucleoprotein (red-brown pigment), counterstained with haematoxylin.

Following inoculation of ten, sixteen week old specific pathogen free (SPF) chickens with 10^7.7^ EID_50_ of live A/Anhui/1/2013 A (H7N9) virus, by the oral/nasal/ocular (ONO) route five chickens succumbed to infection, or were humanely killed between days 4 and 8 PI (**Figure 1A**), exhibiting disease signs consistent with AI (depression, hunched posture, ruffled feathers, drooped wings, head-tucking, inactivity, isolation from other birds). To rule out co-infection by other common poultry pathogens as a confounding factor that may have contributed to the unexpected enhanced pathogenicity of the infection, specimens were tested for a large panel of avian pathogens using relevant diagnostic tests available at the ACDP (**Supplementary Figure 1**), however no evidence of coinfection could be detected. RT-PCR was performed to detect the presence of H7N9 viral RNA and high levels were detected in peripheral tissues such as heart, spleen, kidney and liver as well as tissues associated with the respiratory and gastrointestinal tract (**Figure 1B**). Furthermore, viral antigen was demonstrated by immunohistochemistry examination (**Figure 2**, which showed the presence of antigen in airsac membranes, single cells (possibly macrophages) in multiple tissues (including spleen and other lymphoid tissues, lungs, liver, various mucosa and sub-mucosa), endothelium of blood vessels, principally capillaries in various organs including brain and heart, pancreatic acinar cells, and in epithelium of the upper respiratory and intestinal tracts and kidney tubules (**Figure 2**). Microscopically, the main pathological changes included inflammation of the airsacs, which was associated with the presence of yolk material; moderate to severe diffuse interstitial nephritis, with localized severe interstitial and tubular necrosis; mild to severe inflammation of the upper respiratory tract and associated structures, such as the peri-ocular tissues leading to ocular prolapse in one bird; and in one case, localized necrosis of the pancreas. As these clinical outcomes were unexpected for an LPAI virus such as this strain of H7N9, we further examined the virus recovered from chickens that succumbed to disease to assess any viral determinants that may have contributed to the enhanced disease phenotype observed.

### Changes in the HA and NP correlate with disease pathology in chickens

**Figure 3:**
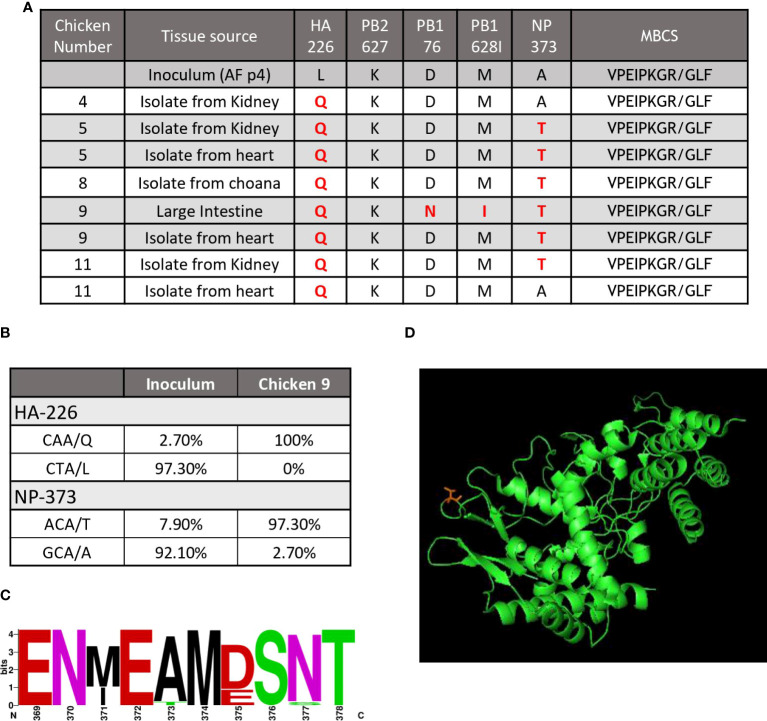
A mutation in the NP correlated with disease outcome. **(A)** The table shows a summary of the results of genome sequencing of virus isolated from H7N9 infected chickens. Key, known mutations, including presence absence of the MBCS associated with pathogenesis are shown. Changes are highlighted in red. **(B)** NGS quantification of two key mutations demonstrates the abundance (shown as percentage) of each mutation from the inoculum and a heart isolate from an infected chicken (chicken #9). **(C)** The sequence logo shows the abundance of different amino acids from available Genbank sequences for aa369-378 of the influenza NP protein **(D)** and predicted protein folding of the influenza NP protein showing amino acid 373 (T) (in red) on a solvent exposed side chain.

In order to determine what, if any, viral mutations had occurred that may have caused the H7N9 virus to cause severe disease in chickens, we performed full genome sequencing on virus re-isolated (by culture in specific pathogen free eggs) from infected tissues including heart, kidney and intestine, from birds that succumbed to disease. A notable H7N9 mutation that was detected in at least one tissue in 4 of the 5 chickens that succumbed to disease, but not in chickens free of clinical signs, was the NP A373T mutation. We did however observe the HA L226Q change which has previously been associated with increased binding affinity for the chicken type α2, 3 sialic acid (**Figure 3A**). Whilst important, this change was also observed in the HA of virus from infected birds that did not succumb to infection or display any clinical disease (data not shown) and is therefore unlikely to be the sole cause of the change in disease phenotype observed. To further examine these changes, we performed next generation sequencing to compare the relative abundance of the HA226 and NP373 mutations in the genome of the virus inoculum to that of virus present in the large intestine of one of the chickens (#9) that succumbed to disease (**Figure 3B**). Our data shows that the original inoculum contained both the HA L226Q as well as the NP A373T SNPs at low abundance (2.7% and 7.9%, respectively), however following infection the resultant amino acids present at these locations were predominantly HA 226Q (100%) and NP 373T (97.3%), suggesting these two mutations had a fitness advantage in the infected chickens. Whilst is has been found that a small proportion of circulating H7N9 contains the HA 226Q (19), we were also able to show that the NP 373T mutation was present, although not common, in viral genomes deposited in GenBank (**Figure 3C**). Interestingly, the region encoding this mutation in the NP is well characterized as an immunodominant T cell epitope (20). Furthermore, we mapped this change to a predicted structure of the NP protein and we were able to demonstrate that it is present on an exposed surface (**Figure 3D**). It has been demonstrated that the NP 360-373 loop is critical in RNA binding (21). Importantly, none of the viral isolates had incorporated multiple basic amino acids in the cleavage site of the HA protein (**Figure 3A**), which is recognized as the major determinant of AI virus pathogenicity. In the large intestine of a single chicken, two additional changes were observed in the PB1 (D76N and M628I), however these changes were not observed in specimens tested from other chickens and were therefore unlikely to be the cause of the change in disease phenotype. Whilst the mutation in the NP protein appeared to correlate with disease, it was important to confirm this empirically.

### NP 373T bearing H7N9 causes severe pathology in IVPI studies

**Figure 4:**
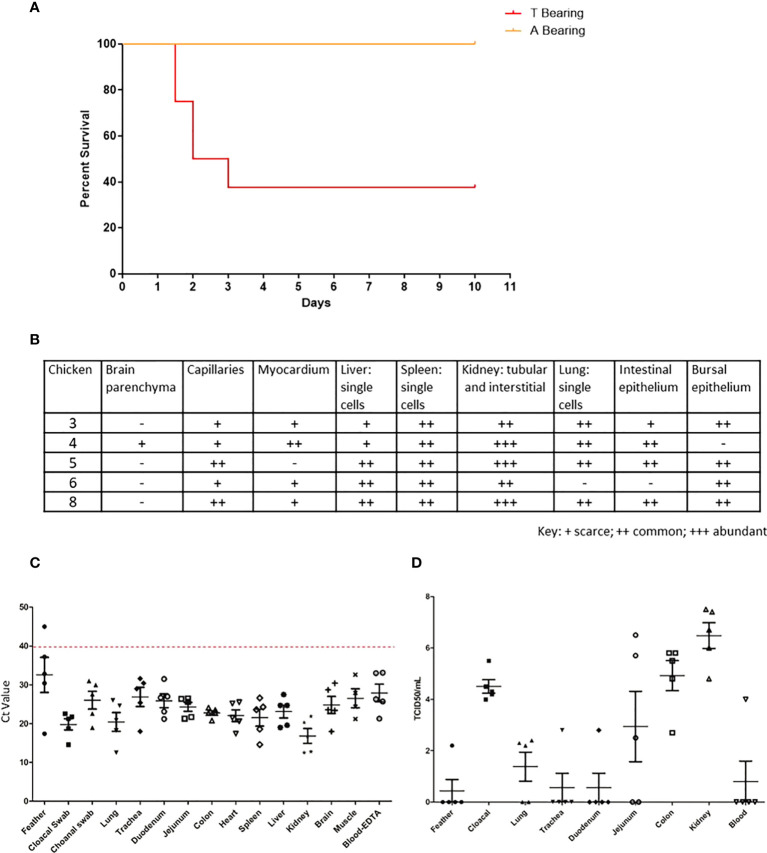
The 373T NP virus isolate causes severe disease and death but not the 373A NP. Survival curve **(A)**, RT-qPCR **(B)** histopathology scores, qPCR **(C)** and live virus detection **(D)**, of H7N9 systemic infection, following intravenous inoculation of SPF chickens with NP 373A and NP 373T bearing variants of A/Anhui/1/13 (H7N9). **(A)** Following inoculation of eight 16-week old SPF chickens with a 1:10 dilution of allantoic fluid containing live A/Anhui/1/13 (H7N9) bearing NP 373T, five chickens were humanely killed between day 2 and 3 pi. In contrast, following inoculation of eight 16-week old SPF chickens with a 1:10 dilution of allantoic fluid containing live A/Anhui/1/13 (H7N9) bearing NP 373A, all eight chickens survived until the predetermined endpoint (day 10 pi). **(B)** histopathology scores are presented for various tissues taken from susceptible chickens. Swab and tissue samples taken upon necropsy from the five chickens infected with the NP 373T bearing variant of A/Anhui/1/13 (H7N9), that were prematurely humanely killed, were subjected to RT-qPCR analysis of the influenza M gene **(C)** and live virus titration in MDCK cells **(D)** to assess the degree of systemic infection in the affected chickens.

**Figure 5:**
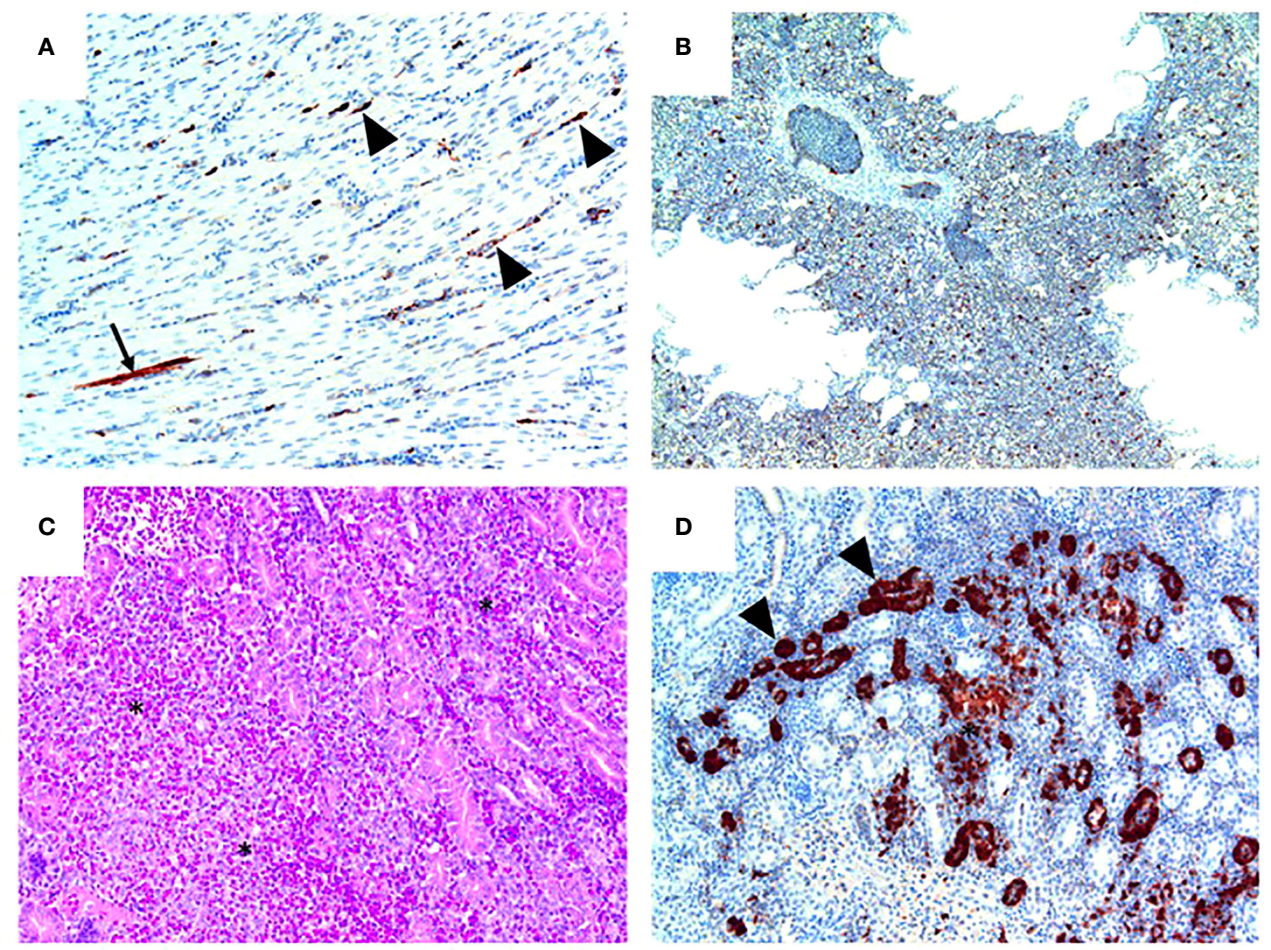
Pathology and H7N9 antigen tropism in chickens inoculated with the NP 373T bearing variant of A/Anhui/1/13 (H7N9) by the intravenous route. **(A)** Heart (chicken 5) showing viral antigen capillary endothelium (arrowheads) and in a single myocardial fibre (arrow); tissue morphology is normal in this heart. **(B)** Lung (Chicken 8) showing viral antigen within single cells; tissue morphology is normal in this tissue. **(C)** Kidney (Chicken 4) showing acute interstitial and tubular necrosis, haemorrhage and histiocytic infiltration (*). **(D)** Kidney (Chicken 4; consecutive section to that shown in **(C)** showing viral antigen in tubular epithelium (arrowheads) and localised necrosis (*). Immunohistochemistry for influenza nucleoprotein (red-brown pigment), counterstained with haematoxylin **(A, B, D)**; haematoxylin and eosin stain **(C)**.

To further determine the role of the NP 373T H7N9 isolate, we performed comparative studies using the IVPI method, whereby eight chickens were inoculated intravenously. We performed these studies with both the NP 373T and NP 373A isolates, both of which were HA 226Q. The chickens infected with the NP 373A isolate survived until the end of the study showing either very mild or no clinical signs of LPAI. Conversely, 5/8 chickens receiving the NP 373T inoculum succumbed to disease (either died or were humanely killed between days 2 and 3 PI. (**Figure 4A**). Upon histopathological assessment of tissue samples from these chickens, viral antigen was detected in multiple tissues of all 5 chickens, suggestive of a systemic infection with virus antigen most prominent in the kidneys (**Figure 4B**). We used qRT-PCR for influenza virus to further analyse samples from the chickens that succumbed to infection and were able to demonstrate low Ct values (high amounts of viral genome) in all tissues tested (**Figure 4C**). We were also able to detect live virus in multiple tissues sampled from the chickens that succumbed to infection, including lung, colon and kidney (**Figure 4D**). In particular it was noted that there was localized acute interstitial and tubular necrosis of the kidneys,which was associated with viral antigen within tubules and necrotic regions, and viral antigen in endothelium of various tissues (e.g. heart, brain, liver) and in single cells (macrophages) of various tissues (particularly lung, liver and lymphoid tissues) (**Figure 5**). Small to moderate amounts of viral antigen were also found in epithelium of bursa and intestine, in foci of glial cells in the brain and in the nasal glands and turbinate epithelium (data not shown). Interestingly, of the 3/8 chickens that did not succumb to disease, all three showed no clinical signs and remained clinically healthy throughout the study. Upon histological analysis of tissues sampled at necropsy, two of these chickens did not show signs of pathological changes in tissues, one had chronic renal and myocardial inflammation, which may have been the outcome of mild viral infection (data not shown). These results suggested that the NP 373T isolate was associated with a more enhanced disease phenotype in a subset of infected chickens. We further sought to understand the impact of the NP A373T mutation on the immune system to determine whether there was a possible correlation with the host immune response following infection with other HPAI viruses.

### H7N9 pathology in chickens is associated with a rapid and specific loss of CD8 T cells

**Figure 6:**
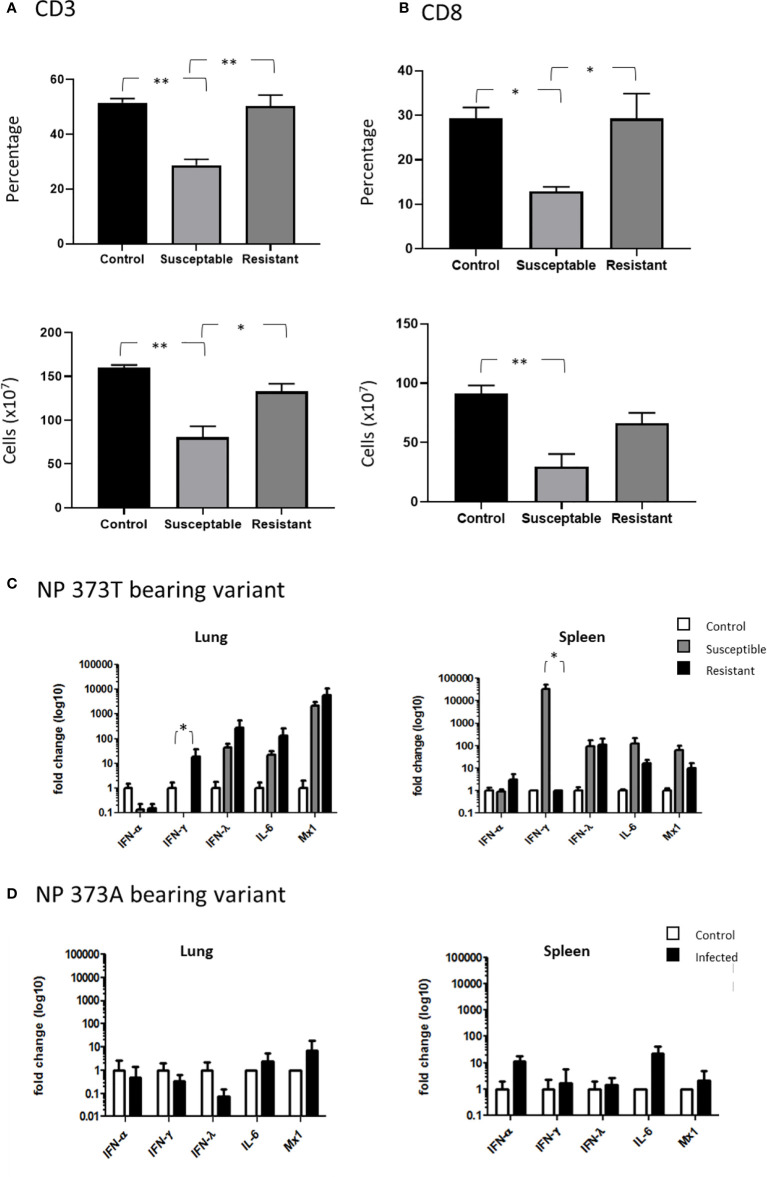
Severe disease is associated with severe CD8^+^ T cell depletion. Flow cytometry analysis of the proportions of **(A)** CD3^+^ T cells and **(B)** CD8^+^ cells comparing healthy uninfected control chickens to susceptible and resistant chickens. Cytokine expression analysis as determined by qPCR, in the lung and spleen of chickens infected with the **(C)** NP 373T bearing variant of A/Anhui/1/13 (H7N9) compared chickens infected with the **(D)** NP 373A bearing variant of A/Anhui/1/13 (H7N9). Error bars represent SEM. *p<0.05, **p<0.01.

In order to further understand the impact of the NP 373T phenotype, we performed an extensive cellular phenotyping of the leukocytes from the spleen of chickens infected with the NP 373T isolate, as pathology associated with AI is commonly associated with leukopenia and lymphopenia. We compared uninfected control chicken spleen samples to both spleen samples from chickens that succumbed to infection (susceptible, humanely killed days 1-3) and those from surviving chickens that remained in the experiment until the predetermined end point at day 10 (resistant). We demonstrated no significant changes in proportions of CD4 T cells, MHCII^+^ cells and Kul1^+^ macrophages as well as no significant changes in the proportions of B cells, MHCII or Vβ1, Vβ2 or ɣδ on CD3^+^ T cells (**Supplementary Figures 2** and **3**). We did however find a significant reduction in the proportion of total CD3^+^ T cells in susceptible chickens when compared to control chickens (**Figure 6A**). Upon further investigation we discovered this loss was restricted to CD8^+^ T cells (**Figure 6B**). Interestingly, no significant change in CD3^+^ or CD8^+^ T cells (**Figures 6A, B**) was observed in resistant chickens compared to controls.

Another hallmark of AI pathology is hypercytokinemia and we therefore undertook cytokine gene expression analysis between control chickens, and those that were susceptible/resistant to the NP 373T isolate (**Figures 6C, D**) We demonstrated that the NP 373T isolate induced a robust increase in IFN-λ, IL-6 and Mx transcription in both the lung and spleen for both susceptible and resistant chickens when compared to uninfected controls. Interestingly, IFN-α transcription remained unchanged in both the lung and spleen for susceptible and resistant chickens. The most striking result was a complete loss of IFN-γ transcript in the lungs of susceptible chickens and a robust increase in transcripts in the spleen. In stark contrast, chickens infected with the NP 373A isolate showed only a modest increase in IL-6 and IFN-α in their spleen (**Figures 6C, D**). These data suggest the NP 373T isolate generates a robust cytokine response when compared to the NP 373A isolate. A question still remains as to why infection with the NP 373T isolate led to increased pathogenicity in 5/8 chickens, while the remaining three chickens displayed no signs of disease. To investigate this further we focused on potential host factors that could lead to increased pathology.

### Susceptible chickens have a SNP in IFITM1

It is well established that host factors play an important role in disease outcome and we therefore sought to identify differential factors leading to high levels of pathology in some chickens but not others. It has been previously suggested that an alternative splicing of chicken MHC is responsible for variable disease outcomes (22), however upon sequencing the relevant region of MHCI we determined all chickens had a complete MHCI transcript (**Supplementary Figure 4A**). In addition to alternate splicing, the MHCI haplotype has been suggested as important in disease susceptibility in birds and mammals (23), and we therefore determined the MHC (B complex) haplotype in the resistant and infected chickens. We demonstrated that 4/5 susceptible chickens presented with the B19 or B21 haplotype either in addition to or in isolation of the B15 haplotype **(Supplementary Figure 4B**). Given the low numbers of chicken we are unable to determine whether the presence of the B19 or B21 alleles correlates with disease outcome. Due to this limitation, we are unable to draw any conclusions on the impact of MHCI haplotype on disease outcome. As SNPs in the IFITM gene family have been previously associated with increased disease susceptibility (11) we investigated the potential for SNPs in the chicken IFITM3 gene to play a role in the H7N9 susceptibility, however all chickens sequenced displayed the wild type transcript for IFITM3 (**Supplementary Figure 5A**). As the annotation and characterisation of chicken IFITM genes is less advanced than other species, we also investigated other chicken IFITM genes and found the presence of two SNPs in exon 1 of the chicken IFITM1 gene that appeared to correlate with disease susceptibility (**Supplementary Figure 5B**). We performed a Fishers exact test on all chickens that had been infected with the NP 373T isolate and found a statistically significant correlation between the presence of either SNP, however the correlation was stronger for the second SNP (p=0.0012 vs p=0.0263) (**Supplementary Figure 5C**).

## Discussion

We analysed the pathology and immune response of chickens infected with H7N9 influenza *via* either the oro/nasal/ocular (ONO) or intravenous routes of infection and observed that in some of the chickens inoculated the disease appeared similar to that caused by highly pathogenic AI viruses despite the absence of a haemagglutinin multi-basic cleavage site within the infecting virus. This provides an excellent opportunity to study the mechanisms of pathology, which may be partially translatable to the disease seen in humans. By examining and dissecting the host pathogen interactions we have been able to determine key viral changes involved in the development of this enhanced disease phenotype. Additionally, we observed distinct differences within cohorts of infected chickens, whereby subsets of chickens succumbed to infection or were humanely killed due to severe clinical disease, and other chickens presented with no clinical signs at all, suggesting host factors were also contributing to disease outcomes, again, similarly to what has been published for human H7N9 infections (24).

Previous reports of chickens infected intranasally with the Anhui/1/2013 H7N9 strain demonstrated no signs of clinical disease and virus was only able to be recovered from the trachea and lung of infected animals, consistent with LPAI infections (9). Similarly, chickens infected *via* the intravenous route with multiple different inoculum concentrations demonstrated a pathogenicity index of 0 (25). This suggests that our initial findings derived from ONO infection of 10 chickens, where 5 succumbed or were humanely killed due to severe clinical disease, were highly irregular for the Anhui/1/2013 viral strain. Co-infections with other microorganisms have been shown to enhance the impact of LPAI infections in chickens, therefore we undertook extensive testing for advantageous agents, however, no co-infections were detected in any of the chickens, suggesting Anhui/1/2013 was the sole causative agent of disease (**Supplementary Figure 1**) (26). However, many of the affected chickens had yolk material within airsacs, and the resulting airsac inflammation may have provided the co-factor necessary in some chickens for viral replication. Clinical signs of disease were consistent with HPAI, including depression, hunched, ruffled feathers, droopy wings, head-tucking, etc., further suggesting involvement of AI. To further ascertain the cause of disease we performed qPCR on swabs and tissues and demonstrated the presence of high levels of viral genome in multiple tissues including heart and spleen. Additionally, histopathology analysis revealed presence of antigen in multiple systemic tissues, including in endothelium and macrophages, as is typical of HPAI infection (27, 28). Such a significant change in disease phenotype, in the absence of co-infection, would suggest changes in viral fitness, including the insertion of a MBCS (29). Following viral genome sequencing from multiple chickens we were unable to detect the presence of a MBCS. This is similar to the disease outcomes of humans infected with H7N9, whereby the virus contains no MBCS but is still able to cause severe disease and death (30). A similar phenomenon has occurred in an outbreak of avian influenza (H3N1) in poultry in Belgium, which resulted in an increase in mortality, viral replication and length of virus excretion in infected birds (31). Therefore, understanding the mechanism of disease progression and pathology seen in chickens infected with this virus may help us further understand how H7N9 infections lead to disease in humans. Although no MBCS could be detected we were able to demonstrate a number of changes in the virus isolated in these chickens. Notably, the HA226L of the A/Anuhi/1/2013 virus, which has previously been attributed to mammalian adaptation through enhanced sialic acid receptor binding, had changed to HA226Q, which has been shown to increase binding to chicken α 2, 3-sialic acid (32). It was unlikely that this change alone led to the increased pathogenicity as many LPAI viruses, including the H7N9 A/Shanghai/1/2013 strain, bear the HA 226Q and have a demonstrated LPAI phenotype (25). Furthermore, viral isolates from a number of the chickens that survived the ONO infection without clinical disease also had the HA L226Q change, suggesting additional modifications may be important. Interestingly, we observed a non-synonymous change in the NP protein (NP A373T), in nearly all chickens that succumbed to disease, but not in surviving chickens. When we compared the inoculum to the virus isolated from one of the susceptible chickens by next-generation sequencing we were able to demonstrate that the HA-L226Q and the NP-A373T changes appear to have arisen from a quasi-species that was present in the inoculum. Our data suggests that both the HA-L226Q and the NP-A373T amino acid changes may have conferred a level of viral fitness as both changes represented less than 10% of the virus inoculum but greater than 90% of the re-isolated virus following infection. Whilst a number of viral fitness mutations have been reported to confer increased pathogenicity to AI in birds and mammals, previous reports have centered on the PB2 and to date few reports have implicated the NP in pathogenicity (33, 34). We performed a meta-analysis of >800 NP sequences from H7N9 and demonstrated that the region from amino acid 371-377, encompassing the NP A373T mutation is highly variable when compared to the remainder of the protein. This may imply a selective fitness in this region.

Upon further investigation in an intravenous inoculation model, we demonstrated a distinct difference in ability to cause disease when we compared a NP-373A isolate to an NP-373T isolate, both of which were HA-226Q. In this study we observed no disease in chickens inoculated with the NP-373A isolate (wild type NP), however 5 of 8 chickens succumbed to disease within 72h following inoculation with the NP-373T isolate. This is further evidence that the NP-A373T amino acid change has played a role in increasing the pathogenesis of the H7N9 virus in chickens. It was notable that these infections resembled a highly pathogenic phenotype with a short progression time, high viral genome and antigen levels in systemic tissues, as well as typical signs associated with avian influenza infection in chickens. The NP has been most commonly associated with immunodominant CD8^+^ T cell stimulating epitopes in multiple species, and in particular, the region encompassing this change is a well-documented mouse immunodominant peptide (NP 366-374) (35). To this end we investigated the impact on leukocyte populations in susceptible chickens. When we compared the proportions and total cellularity of a range of leukocytes we only observed significant differences in the total CD8 populations and consequently in the CD3 population as compared to uninfected control chickens from the same cohort. This significant reduction was not observed in the chickens with no apparent clinical disease, and furthermore, there were no significant changes in other lymphocyte or myeloid populations tested. Previous studies in mice have also implicated variations in NP peptide presentation in alternative activation of T cells resulting in differential disease outcome. Ream et.al., (2010) demonstrated that peptides with only two amino acid substitutions can lead to changes in pathology through increased apoptosis of CD8 T cells (36). This was suggested to be due to a loss in expression of the prosurvival molecules, Bcl-2 and Bcl-xL, possibly through changes in TCR-peptide avidity. Therefore, the changes we have observed in the NP may have led to increased pathology through changes in peptide/MHCI-TCR interactions. In addition to the impact on T cell subtypes, the mutation in the NP protein it has been demonstrated that the NP 360-373 loop is critical in RNA binding (21). This role in the stabilization of RNA is an essential role of NP in replication of the virus and may lead to increased proliferation potential. Further investigation is required to determine if a mutation of NP 373A to 373T is capable of leading to increased disease severity.

Interestingly, of the chickens that did not succumb to disease, we observed no clinical signs of illness, creating a stark contrast to chickens that had severe clinical progression, and raising the question about the possible mechanisms of resistance/susceptibility.

Previous studies of influenza susceptibility have focused on host elements, typically of the immune system (37). In particular, the IFITM3 molecule has been shown to be important in response to influenza infection and implicated in increased susceptibility through a single nucleotide polymorphism (SNP) in the IFITM3 gene, *rs12252-C* (38). This SNP has been correlated with worsened clinical outcomes for both seasonal influenza and H7N9 infections (11). When we examined the sequence of the IFITM3 gene as described by Smith et al. (2013) (39) and confirmed more recently by Bassano et al. (2017) (40) and despite an annotated presence of potential SNPs in the gene we detected no differences between susceptible and resistant chickens. We did however detect two SNPs in chicken IFITM1 which had a significant correlation with disease susceptibility (p=0.0263 and p=0.0012 for the first and second SNPs respectively).

## Conclusions

Here we present the first report of H7N9 adapting to an increased pathogenicity phenotype (in chickens) without incorporating an MBCS, and propose mutations in the NP to be causing these changes in phenotype. Furthermore, it appears a SNP in an IFITM gene may play a key role in susceptibility. As the H7N9 infections have caused a significant impact on human health, the ability of this mutation to facilitate enhanced virus pathogenicity for chickens warrants further investigation into the role it plays in determining influenza virus pathogenicity in the human population.

## Methods

### Ethics

All animal studies were approved by the CSIRO Australian Centre for Disease Preparedness (ACDP) Animal Ethics Committee (permit numbers 1687 and 1775) and conducted following the Australian Government National Health and Medical Research Council, Australian code for the care and use of animals for scientific purposes.

### Virus

Influenza virus A/Anhui/1/2013 (H7N9), isolated from a human in China, and obtained from the World Health Organisation Collaborating Centre for Reference and Research on Influenza (Melbourne), was used in this study. Virus was propagated by allantoic cavity inoculation of 9–11-day-old specific pathogen free (SPF) embryonated chicken eggs. The virus stock was titrated in chicken eggs and the 50% egg infectious dose (EID_50_)/mL was calculated according to the method of Reed and Muench (41). All *in vitro* and *in vivo* work involving live H7N9 virus was conducted within biosafety level 3 facilities at the ACDP. Animal work was performed using full protective clothing and powered air purifying respirators.

### Animal studies

SPF chickens (obtained from Australian SPF services) were used for each of the animal experiments. Prior to challenge, serum was collected from each chicken to confirm that all chickens were serologically negative for avian influenza A virus, as determined by blocking ELISA (42). Following virus inoculation chickens were observed closely from 22 hpi and humanely killed once they reached the humane endpoint, defined as progression to moderate signs of disease, including facial swelling, diarrhoea, hunched posture with ruffled feathers, drooping wings, huddling, recumbency, depression and slow response to stimulation. Chickens were humanely killed by cervical dislocation following heart bleed under anaesthesia (ketamine 44 mg/kg, xylazine 8 mg/kg injected intramuscularly). For the ONO study, ten, 16-week-old chickens were inoculated with 7.7 Log_10_EID_50_ of H7N9 virus diluted in a 0.5 ml volume of PBS, by dropwise addition of the inoculum spread evenly between the eyes, nose and throat. For the intravenous pathogenicity index (IVPI) studies, eight, 4- to 8- week-old chickens were intravenously inoculated into the jugular/wing vein with 0.2 ml of H7N9 infected allantoic fluid, diluted 1:10 in PBS. Back titration of both viral inoculums was performed showed that the NP 373T bearing isolate was administered at 8.0 Log_10_ EID50/0.2ml inoculum and the NP 373A bearing isolate was administered at 8.3 Log_10_ EID50/0.2 ml inoculum. Immediately following humane killing, oral and cloacal swabs and blood and tissue samples were taken from all chickens. Swabs were placed into PBS containing antibiotics (100 units/mL penicillin (JRH Biosciences), 100 μg/mL streptomycin (Sigma) and 50 μg/mL gentamycin (Sigma)). Blood samples were collected in serum clotting and EDTA tubes, and approximately 100 mg of each tissue sample was collected into sterile 2 mL tubes containing PBS with antibiotics and a small quantity of 1 mm silicon carbide beads (BioSpec Products). Tissue samples were homogenized once for 20 s in a FastPrep24 tissue homogenizer (MP Biomedicals) for virus titration. Comparative tissue samples were also collected in 10% neutral buffered formalin and in PBS for histopathological and immunological analyses, respectively.

### Analysis for common poultry pathogens

Various chicken homogenised tissue and oral swab samples were subjected to routine ACDP diagnostic assays in order to exclude the presence of the following pathogens: chicken anaemia virus, infectious bursal disease virus, avian metapneumovirus, Ornithobacterium rhinotracheale, infectious laryngotracheitis virus, infectious bronchitis virus, Newcastle disease virus, H5 subtype Avian influenza virus, mycoplasma and Salmonella (detailed methods available upon request).

### Virus detection

The presence of influenza viral genome within swabs, tissues and blood samples was assessed by extracting the total RNA from each sample (MagMax-96 Total RNA Isolation Kit, Life Technologies) and analysing the extracted RNA using a pan-influenza A matrix gene real-time RT-PCR assay (43). Live virus titrations were performed on Madin-Darby canine kidney (MDCK;ATCC #CCL-34) cell monolayers in 96-well microtitre plates as described previously (10).

### Histology and immunohistochemistry

Histological analysis of chicken tissues following infection was performed as described previously (27). Tissues were fixed in 10% neutral-buffered formalin for 24 h, processed into paraffin wax, cut and stained using haematoxylin and eosin for examination of histopathological lesions. Consecutive tissue sections were also stained in an immunohistochemistry test for influenza A virus nucleoprotein (27).

### Genome sequencing

Viral RNA was extracted from swab and tissue samples and subjected to influenza genome sequencing as previously described (44). In order to quantify mixed amino acid proportions at relevant SNPs, paired-end reads were trimmed in Geneious Prime using BBDuk Trimmer for quality, prior to mapping to A/Anhui/1/2013 (H7N9) reference sequence. SNPs within each codon were identified using the “find variation/SNP tool in Geneious Prime with default settings.

### Virus re-isolation from tissues

Selected tissue samples were homogenised and 200 µl aliquots of each homogenate inoculated into SPF chicken eggs. All eggs were incubated at 37°C with monitoring for up to 5 days post inoculation. The allantoic fluid was then harvested and the presence of influenza virus in the allantoic fluid isolate confirmed by hemagglutination with chicken erythrocytes. The live virus titre (EID50/ml) was determined by inoculating serial log10 dilutions (in PBS) of each isolate into the allantoic cavity of 9-11 day old embryonated chicken eggs. The infectious titre of each isolate was then calculated according to the method of Reed and Muench (1938).

### Genbank SNP prevalence analysis

Sequences were downloaded from Genbank using biopython and aligned using Clustal Omega (version 1.2). Subalignments containing the region of interest were extracted using biopython and saved in fasta format. Sequence logos were created using Weblogo (https://weblogo.berkeley.edu).

### Fluorescence-activated cell scanning

Spleens were cleaned of any connective tissue and mechanically digested in cold FACS buffer (2% (v/v) foetal calf serum, 0.02% (v/v) sodium azide in PBS) to produce a single cell suspension. Mechanical digestion was achieved by pressing the spleen through a 70-μm sieve (BD Biosciences). Cells were diluted to 20 mL in FACS buffer and layered gently over a Lymphoprep density gradient (Axis-Shield) and centrifuged for 20 min at 1000 x *g* at room temperature with no brake. The interphase was collected and washed in 10 mL FACS buffer, centrifuged for 5 min at 400 x *g* before being resuspended in 10 mL FACS buffer. Approximately 10^6^ cells from the spleen single cell suspensions were incubated for 30 min at 4°C in the dark with the following fluorochrome-conjugated anti-chicken antibodies: anti-CD3 fluorescein isothiocyanate (FITC) (clone CT3), anti-CD8a-Cy5 (clone CT8), and anti-CD4 R-phycoerytherin (Pe) (clone CT4) from PickCell Laboratories. All antibodies were diluted in cold FACS buffer. Following incubation, cells were washed in 150 μL of FACS wash and centrifuged at 400 x *g* for 3 min. Cells were resuspended in 150 μL of FACS buffer for flow cytometric analysis. Data was acquired on a BD LSRFortessa X-20 flow cytometer (BD Biosciences) equipped with 405, 488, 561 and 633 nm excitation lasers in conjunction with FACS Diva acquisition software (BD Biosciences). Compensation was performed with single colour using the same conjugated antibodies used in the study. Data analysis was performed using FlowLogic FCS analysis software (Inivai Technologies).

### Cytokine gene expression analysis

qRT-PCRs were performed using a 20x gene expression assay mix (Applied Biosystems) for genes of interest. GAPDH was used for normalization of results. The reactions were performed in MicroAmp Fast Optical 96-Well Reaction Plates (Applied Biosystems). Every reaction was performed in duplicate and contained: 10 µLTaqMan™ Universal PCR Master Mix, 7 µL nuclease free water 1 µL of primer set and 1 µL of cDNA. The reactions were run on the Step One Plus Real-Time PCR System (Applied Biosystems). The amplification program profile was: (50°C for 2 min, 95°C for 10 min followed by 40 cycles of 95°C for 15 sec and 60°C for 1 min). Threshold cycle numbers (Ct) were determined with Sequence Detector Software (Applied Biosystems) and transformed using the ΔCt or ΔΔCt methods using GAPDH as the normaliser gene. The qRT-PCR primers used in this study are available upon request.

### Determination of MHC B complex haplotype

The MHC haplotypes were determined by genotyping the LEI0258 microsatellite locus by PCR-based fragment analysis. Previous studies have demonstrated the genetic sequence of this microsatellite correlates with the MHC haplotype (45). Briefly, genomic DNA was isolated from spleen samples using the QIAamp^®^ DNA Mini Kit (Qiagen GmbH, Hilden, Germany) according to the manufacturer’s instructions. Amplification of the LEI0258 locus was performed using the primers [f]: 5′-ACGCAGCAGAACTTGGTAAGG-3′ and [r]: 5′-AGCTGTGCTCAGTCCTCAGTGC-3′. The haplotype was determined following size and sequence analysis.

### Sequencing of IFITM3 and IFITM1

#### PCR product purification

DNA PCR products, either from excised bands or directly from PCR amplification, were cleaned using a gel and PCR clean up system kit (Promega). Briefly, the gel bands were weighed before the addition of the membrane binding solution at a 1:1 ratio and dissolved in this liquid before continuing with spin and wash steps as per the manufacturer’s instructions. The DNA was eluted from the membrane in 20 µL, 30 µL or 50 µL of nuclease free water (Promega) depending on desired concentration. DNA was stored at -20°C. Full cycle sequencing was performed by the Micromon sequencing facility (Monash University).

#### Cloning and sequencing PCR products

The cloning of PCR products was carried out using the Promega pGEM^®^-T and pGEM^®^-T Easy Vector Systems kit as per the manufacturer’s instructions. The following reagents were vortexed as required and added to a 0.5 mL tube (Eppendorf): 1 µL 10 X Rapid Ligation Buffer, 1 µL pGEM^®^-T Easy (A1360), 1 µL T4 DNA Ligase and 7 µL of PCR product. Pre-made LB Agar gel (200 mL) was melted and allowed to cool for 10 minutes before 200 µL of carbenicillin at a concentration of 100 µg/mL (Sigma) was added, and gently mixed into the gel solution. Within a flow cabinet approximately 25 µL of liquefied gel was poured into sterile 9 cm petri dishes and allowed to set. 50 µL JM109 high efficiency competent cells (Promega) were thawed for 5 minutes on ice before the addition of 2 µL of ligation reaction. The tubes were gently mixed and incubated on ice for a further 10 minutes. The cells were heat shocked in a flowing water bath (Julabo) at 42°C for 45 seconds before being immediately placed on ice for 2 minutes. Room temperature SOC medium (950 µL) was added to the ligation reaction transformations before spinning at low speed using a tube rotator (Ratek) at 37°C for 1 hour. After incubation 100 mL of each transformation culture were added to the pre-set LB agar plates. A lawn was created using a sterile bacterial spreader to evenly distribute the cells. The plates were placed in a hot room at 37°C and left to incubate overnight. The plates were stored at 4°C. Carbenicillin solution (100 µL) at a concentration of 1 g/10 mL was added to 50 mL of LB broth. Aliquots of 5 mL antibiotic LB broth were added to 50 mL tubes (Falcon) before the addition of a single colony to the mix. This was achieved by scraping a single isolated colony from the LB agar plate and ejecting the tip into the 5 mL of broth. The broth solutions were put in orbital shaker overnight at 37°C at 250 rpm. The Qiaprep Spin Mini Prep kit (Qiagen) was used to extract the DNA from1.5 mL of culture, prior to sequencing. Sequencing was performed by the Micromon sequencing facility (Monash University).

#### cDNA synthesis

cDNA was synthesised using a First-Strand Synthesis kit (Invitrogen SuperScript III). The following reagents were combined on ice in a 0.2 mL thin walled PCR tube up to 8 µL as per the manufacturer’s specifications: 6 µL RNA, 1 µL 50 µM oligo[dT]20 and 1 µL annealing buffer. Incubation occurred at 65°C for 5 minutes before the reaction was removed and immediately placed on ice. The following additional reagents were then added to each tube: 10 µL 2 X first-strand reaction mix and 2 µL SuperScripttm III/RNaseOUTtm Enzyme Mix. This mixture was incubated at 50°C for 50 minutes followed directly by a temperature increase to 85°C for 5 minutes before returning the sample to ice. The cDNA product was stored at -20°C.

#### RNA extraction

RNA extraction was performed on cell samples using the RNeasy plus mini kit (Qiagen). Cell lysates (see cell stimulation) were thawed and homogenised before extraction. gDNA was eliminated with the provided columns before the addition of 350 µL of 70% ethanol, equal to the volume of RLT buffer, to the flow through. The sample was washed and dried as per the manufacturer’s instructions (including optional drying step) before elution in 20 µL of nuclease free water (Promega). RNA concentration and purity was determined by NanoDrop (Biolab).

### Statistical analysis

To determine the significant difference between populations of immune cell subsets a One-way ANOVA was performed. Error bars represent the standard error of the mean (SEM). To determine the correlation between disease outcome and presence of the IFITM SNP we performed a Chi-squared test with Yates correction. Alpha for all tests was set at 0.05 and results were considered significant if p values of less than 0.05 were obtained.

## Data availability statement

The data presented in the study are deposited in the Genbank repository, accession number SUB12077964 (sequences OP482185-OP482238).

## Ethics statement

The animal study was reviewed and approved by A CSIRO Australian Centre for Disease Preparedness (ACDP) Animal Ethics Committee (permit numbers 1687 and 1775).

## Author contributions

DSL, JBu, CS, VS, JP, CR, CD, SW, SS, TJG, CC, KB, JW, FW and JBi conducted experimental investigations, DSL, JBu, KK, JBi and DTW provided experimental design and drafted the manuscript. All authors contributed to the article and approved the submitted version.

## Funding

KK was supported by the NHMRC Leadership Investigator Grant to KK (1173871).

## Conflict of interest

The authors declare that the research was conducted in the absence of any commercial or financial relationships that could be construed as a potential conflict of interest.

## Publisher’s note

All claims expressed in this article are solely those of the authors and do not necessarily represent those of their affiliated organizations, or those of the publisher, the editors and the reviewers. Any product that may be evaluated in this article, or claim that may be made by its manufacturer, is not guaranteed or endorsed by the publisher.

## Supplementary material

Supplementary Figure 1 Susceptible birds were not coinfected with common avian pathogens. The inoculum and tissues from susceptible birds were tested for a range of common avian pathogens to determine possible co-infections. These included Taqman PCR (TQM) or conventional PCR (PCR) for H5 variant of influenza (H5), chicken anaemia virus (CAV), infectious bursal disease virus (IBDV), infectious laryngotracheitis virus (ILTV), New Castle Disease virus (NDV), Mycoplasma (Myco), infectious bronchitis virus (IBV) and Salmonella. Neg refers to a negative result.

Supplementary Figure 2 Severe disease is not associated with changes in CD4, Vβ1, Vβ2 or γδ T cells. Flow cytometry analysis of the proportions and total cell numbers in the spleen of **(A)** CD4^+^ T cells, **(B)** Vβ1^+^ T cells, **(C)** Vβ2^+^ T cells and **(D)** γδ T cells comparing healthy uninfected control chickens to susceptible and resistant chickens. Error bars represent SEM. * p<0.05, ** p<0.01

Supplementary Figure 3 Severe disease is not associated with changes in Bu1^+^ B cells or MHCII expression. Flow cytometry analysis of the proportions and total cell numbers in the spleen of **(A)** Bu1^+^ B cells and **(B)** MHCII expressing cells, comparing healthy uninfected control chickens to susceptible and resistant chickens. Error bars represent SEM. * p<0.05, ** p<0.01

Supplementary Figure 4 Relationship between MHCI splicing and haplotype. The agarose gel images show the **(A)** PCR product of exon 7 of chicken MHCI and **(B)** the PCR product of the LEI microsatellite for chickens 1-8. Band sizes were relative to a molecular weight marker MW. Based on size and sequence the LEI PCR product the haplotype has been annotated on the corresponding band. * denotes chickens that succumbed to disease.

Supplementary Figure 5 SNPs in IFITM1 correlate to disease outcome. The ensembl sequence for chicken IFITM3 **(A)** and IFITM1 **(B)** are show with the corresponding sequences of chickens 1-8. Known SNPs are highlighted in red on the ensembl sequence. IFITM1 SNPs in chickens 1-8 are highlighted in red rectangles. The SNPs 1 and 2 summary for chickens 1-8 **(C)** are presented as a table along with disease outcome (susceptible or resistant).
