# Supplementary figures and images for: H7N9 bearing a mutation in the nucleoprotein leads to increased pathology in chickens
Source: Front Immunol. 2022 Oct 6;13:974210. doi: 10.3389/fimmu.2022.974210 (PMC9583263; doi:10.3389/fimmu.2022.974210)

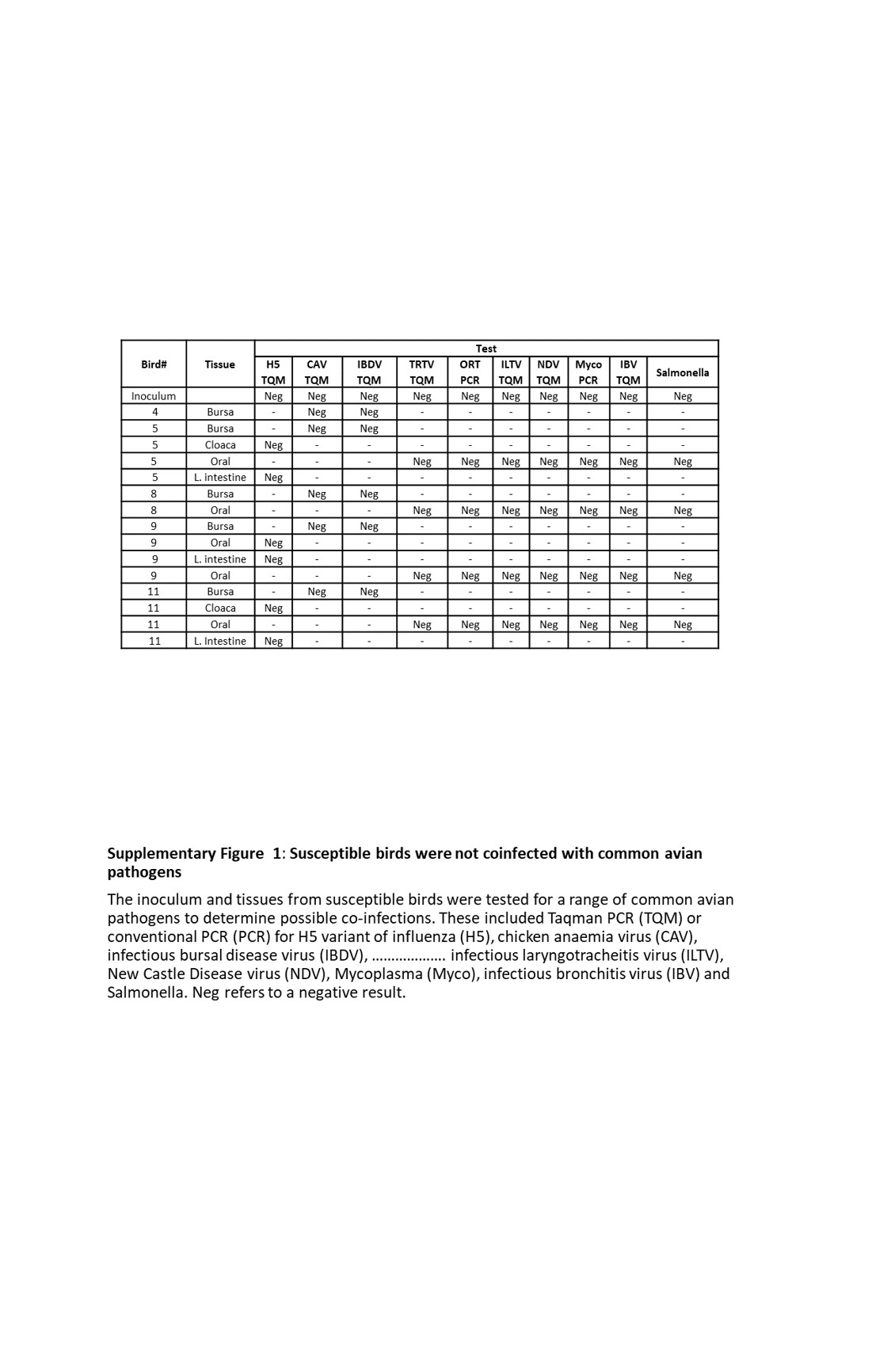

Supplement: Supplementary Figure 1 — Susceptible birds were not coinfected with common avian pathogens. The inoculum and tissues from susceptible birds were tested for a range of common avian pathogens to determine possible co-infections. These included Taqman PCR (TQM) or conventional PCR (PCR) for H5 variant of influenza (H5), chicken anaemia virus (CAV), infectious bursal disease virus (IBDV), infectious laryngotracheitis virus (ILTV), New Castle Disease virus (NDV), Mycoplasma (Myco), infectious bronchitis virus (IBV) and Salmonella. Neg refers to a negative result. [file Image_1.jpeg]

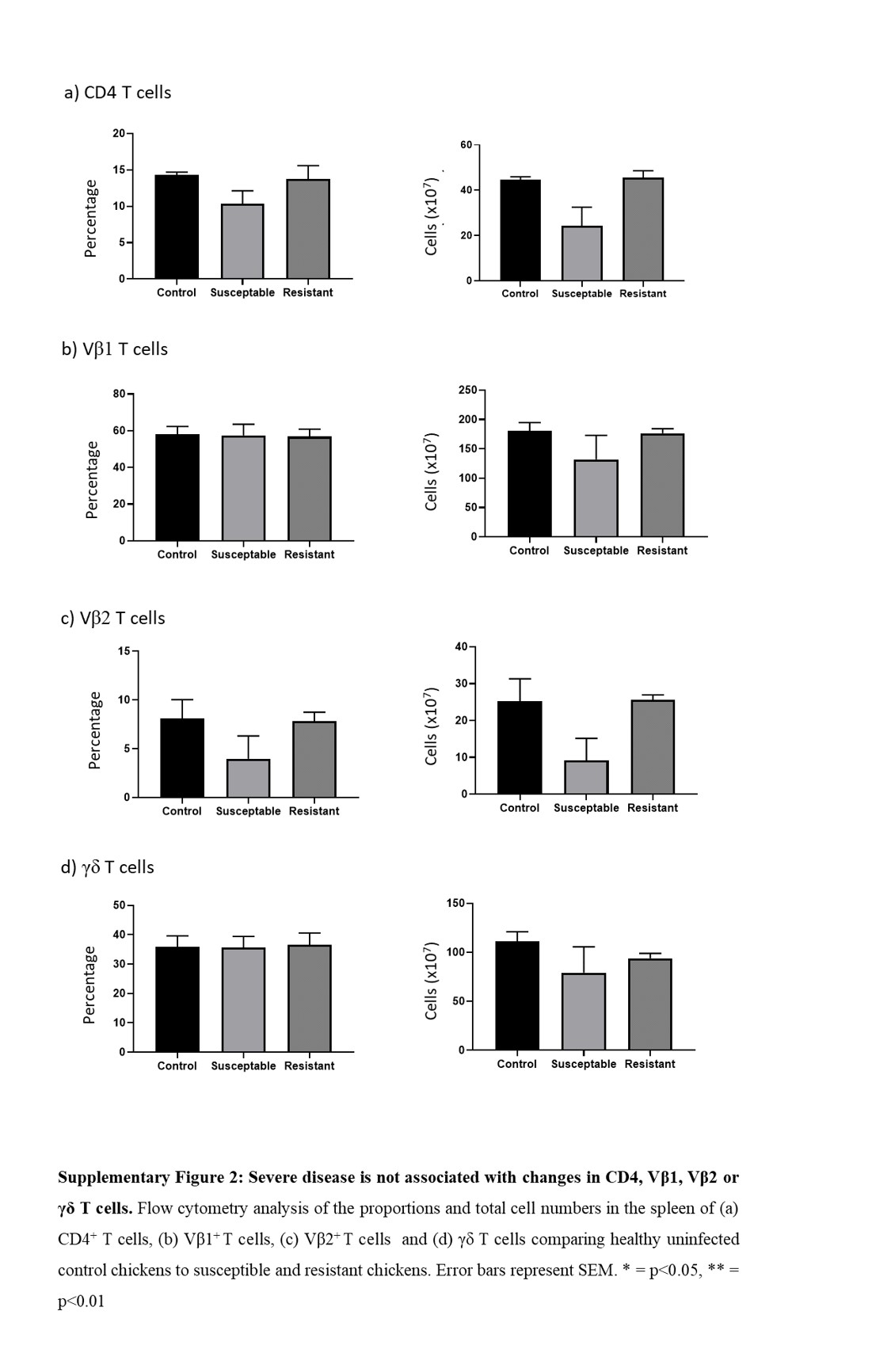

Supplement: Supplementary Figure 2 — Severe disease is not associated with changes in CD4, Vβ1, Vβ2 or γδ T cells. Flow cytometry analysis of the proportions and total cell numbers in the spleen of (A) CD4+ T cells, (B) Vβ1+ T cells, (C) Vβ2+ T cells and (D) γδ T cells comparing healthy uninfected control chickens to susceptible and resistant chickens. Error bars represent SEM. * p<0.05, ** p<0.01 [file Image_2.jpeg]

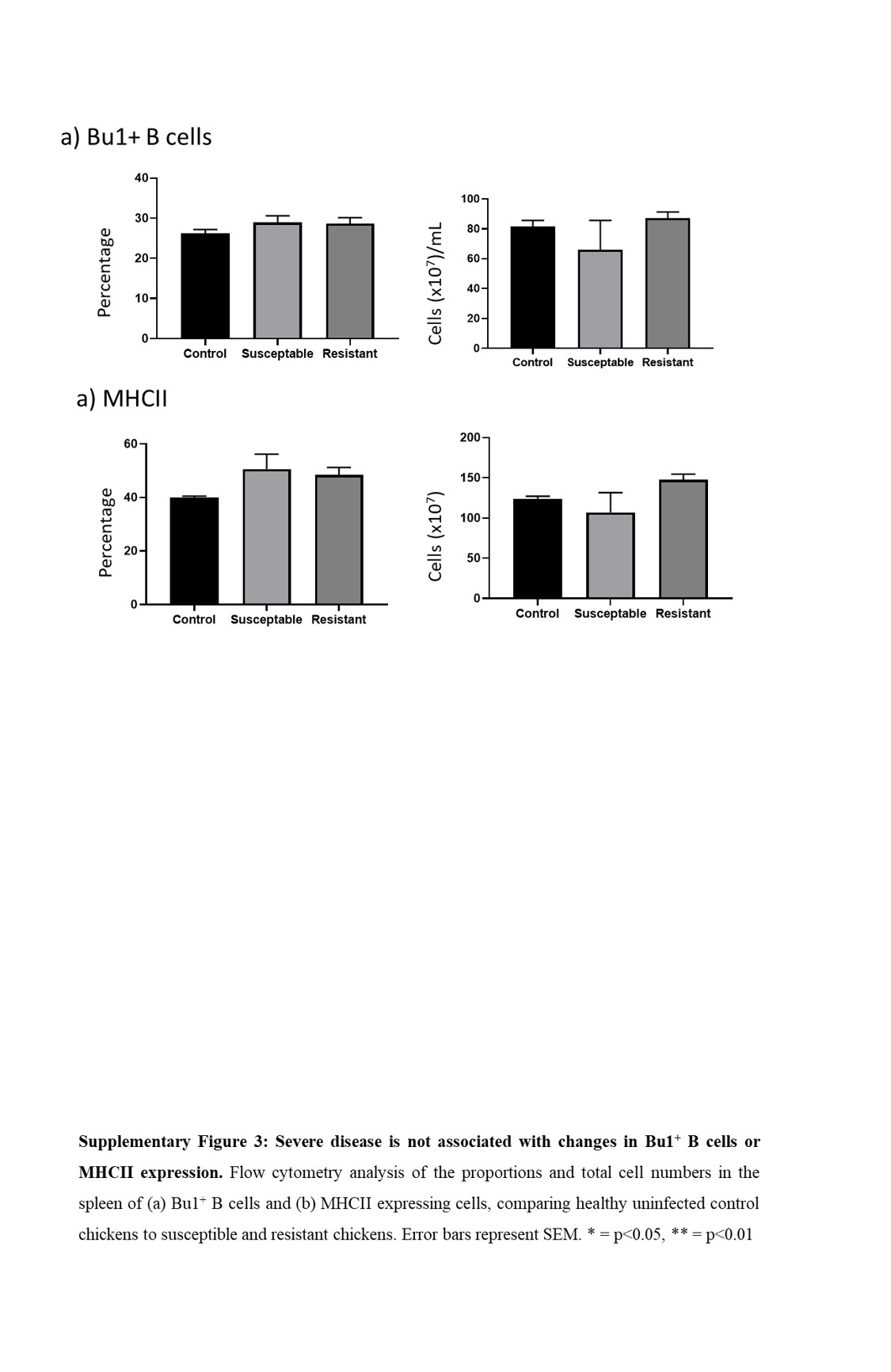

Supplement: Supplementary Figure 3 — Severe disease is not associated with changes in Bu1+ B cells or MHCII expression. Flow cytometry analysis of the proportions and total cell numbers in the spleen of (A) Bu1+ B cells and (B) MHCII expressing cells, comparing healthy uninfected control chickens to susceptible and resistant chickens. Error bars represent SEM. * p<0.05, ** p<0.01 [file Image_3.jpeg]

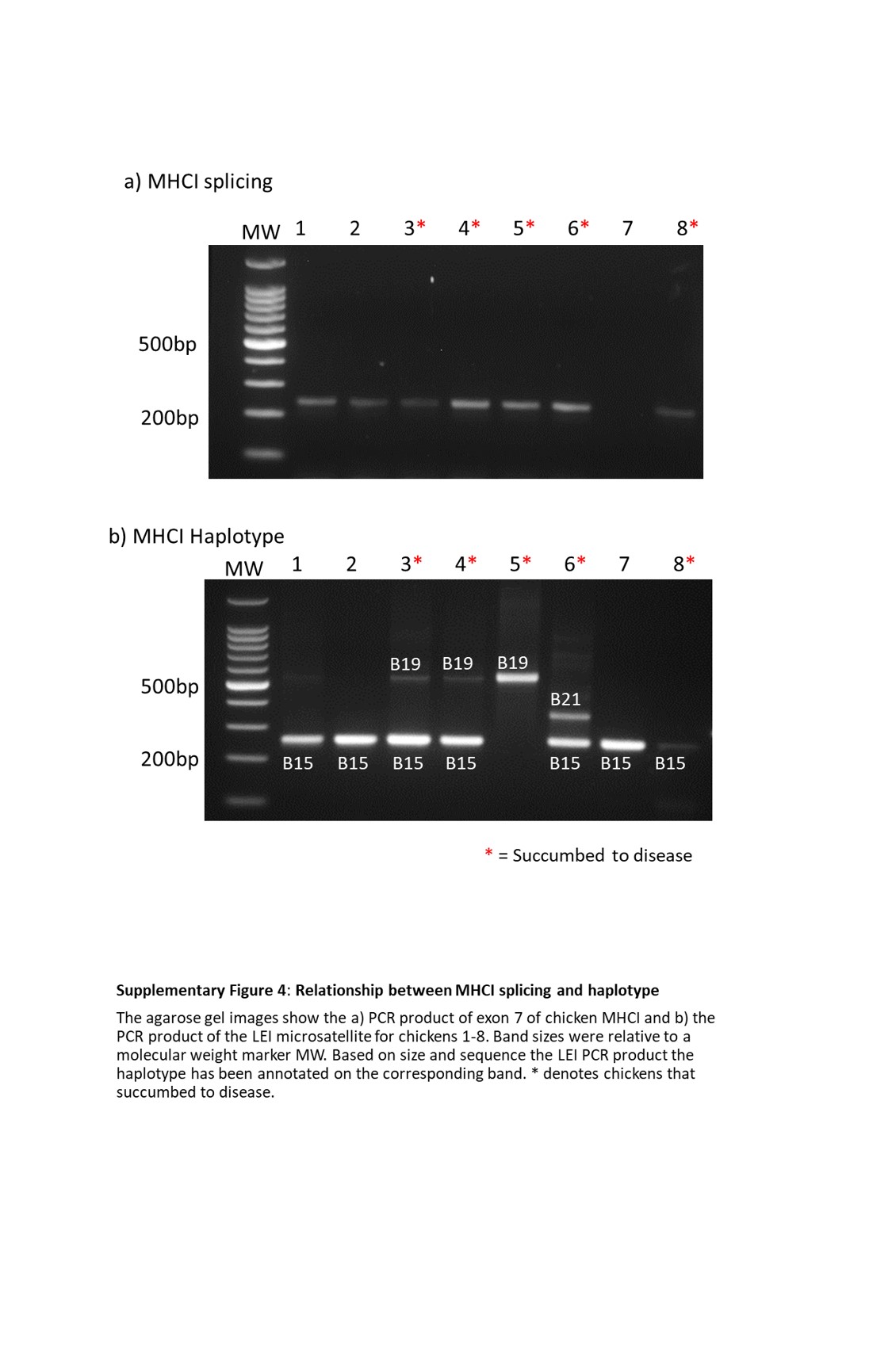

Supplement: Supplementary Figure 4 — Relationship between MHCI splicing and haplotype. The agarose gel images show the (A) PCR product of exon 7 of chicken MHCI and (B) the PCR product of the LEI microsatellite for chickens 1-8. Band sizes were relative to a molecular weight marker MW. Based on size and sequence the LEI PCR product the haplotype has been annotated on the corresponding band. * denotes chickens that succumbed to disease. [file Image_4.jpeg]

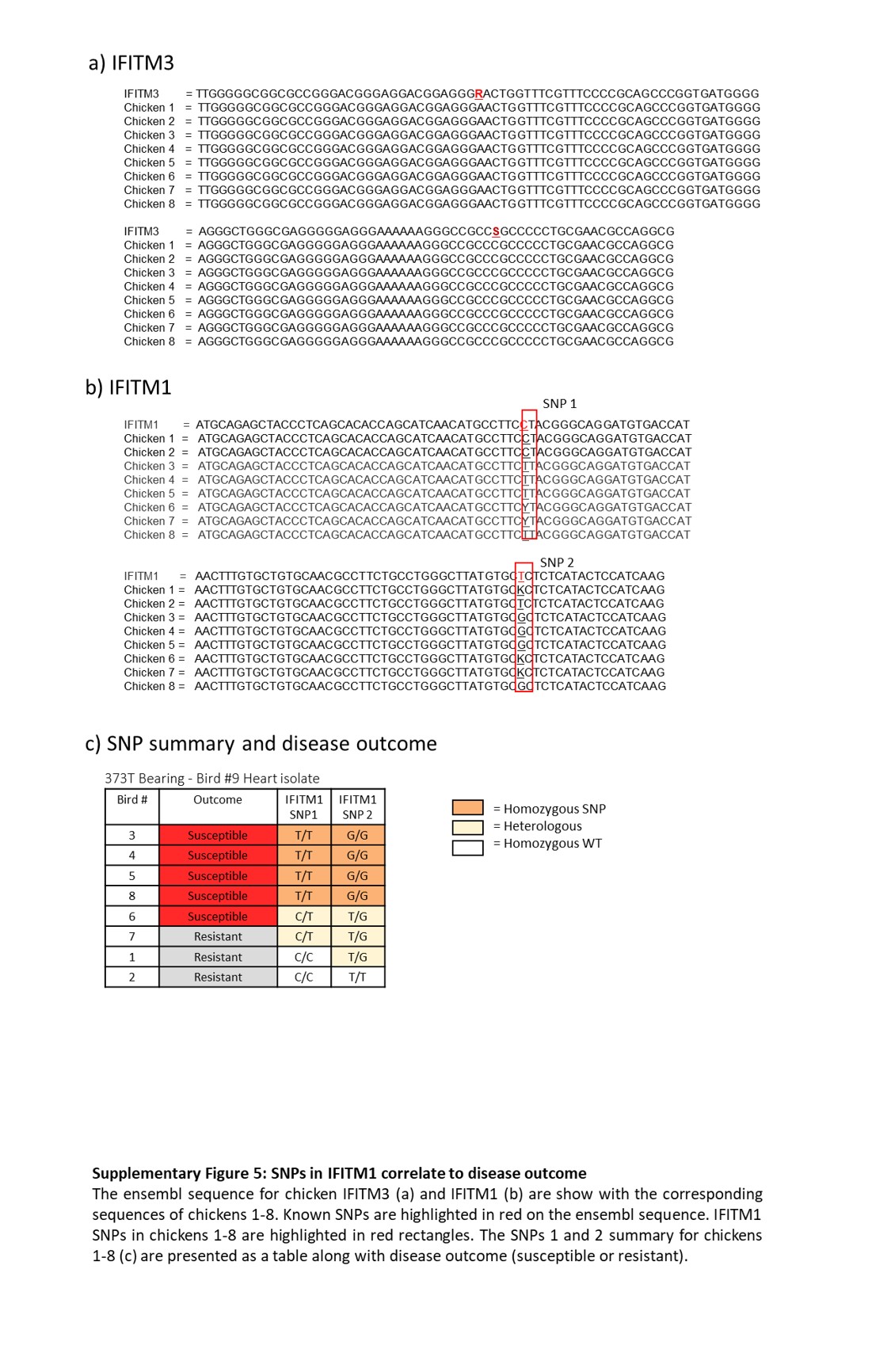

Supplement: Supplementary Figure 5 — SNPs in IFITM1 correlate to disease outcome. The ensembl sequence for chicken IFITM3 (A) and IFITM1 (B) are show with the corresponding sequences of chickens 1-8. Known SNPs are highlighted in red on the ensembl sequence. IFITM1 SNPs in chickens 1-8 are highlighted in red rectangles. The SNPs 1 and 2 summary for chickens 1-8 (C) are presented as a table along with disease outcome (susceptible or resistant). [file Image_5.jpeg]
